# The Cost-Effectiveness of Non-Drug Interventions That Reduce Nursing Home Admissions for People With Dementia

**DOI:** 10.1093/geroni/igab046.877

**Published:** 2021-12-17

**Authors:** Eric Jutkowitz, Fernando Alarid-Escudero, Peter Shewmaker, Joseph Gaugler, Laura Pizzi

**Affiliations:** 1 Brown University, Brown University, Rhode Island, United States; 2 Center for Reserach and Teaching in Economics, Aguascalientes, Aguascalientes, Mexico; 3 Brown Universtiy, Brown University, Rhode Island, United States; 4 University of Minnesota, Minneapolis, Minnesota, United States; 5 Health Outcomes, Policy, and Economics (HOPE) Program, Pscataway, New Jersey, United States

## Abstract

Although people generally want to age in their community, individuals living with dementia are likely to move to a nursing home. In randomized trials, psychosocial interventions reduce the risk of people living with dementia transitioning to a nursing home, but the cost-effectiveness of these interventions is unknown. We used an evidence-based mathematical model to simulate a place of residence (community or nursing home) for people living with dementia. Our model also predicts time caregiving, health care costs, and quality of life. We modeled the reduction in nursing home rate (i.e., hazard ratio (HR) treatment effect) identified from two trials of non-drug interventions for people living with dementia and their caregiver. Using trial data, we account for the disease stage of when interventions are implemented. Specifically, we modeled MIND (HR: 0.63; 18-month effect), an in-home intervention for people with mild-moderate dementia, and the NYU Caregiver Intervention (HR: 0.53; 42-month effect), which is for people with moderate dementia. We evaluated each intervention’s cost-effectiveness relative to usual care for the duration of the intervention from a societal perspective. The MIND and NYU Caregiver Intervention resulted in $23,900, and $6,600 costs savings relative to usual care, respectively. The model predicted an improvement in the quality of life for people living with dementia for both interventions. The largest cost saving was attributed to reductions in family nursing home spending. Medicare and Medicaid received modest cost savings but are likely to be tasked with paying for these interventions.

